# Behind the Placement: A Literature Review of Depression Among Foster Youth

**DOI:** 10.7759/cureus.105482

**Published:** 2026-03-19

**Authors:** Livia Hochman, John Naliyath

**Affiliations:** 1 Psychiatry and Behavioral Sciences, Florida State University College of Medicine, Fort Pierce, USA

**Keywords:** depression, foster care, foster children, major depressive disorder, mental health

## Abstract

Foster children represent a uniquely vulnerable population, with exposure to early adversities predisposing them to psychiatric morbidity, notably depression. Although research has made significant progress in recent years, depression in foster care remains an underrecognized and undertreated disorder. The purpose of this narrative review is to synthesize what is presently known about the prevalence, risk factors, mechanisms, treatments, and remaining gaps in knowledge regarding depression among foster children, with the goal of increasing clinical awareness to assist with screening and intervention, and identifying future research opportunities.

## Introduction and background

Childhood and adolescent depression represent a serious public health concern, since it has wide-ranging and potentially long-term consequences for an individual's psychosocial development and overall quality of life. Children in foster care are at a disproportionately higher risk of developing depression than non-foster children due to a variety of factors, including, but not limited to, familial disruption, trauma, instability, and systemic issues [1-3]. Children and adolescents removed from their homes and placed in alternative care settings often experience significant mental health vulnerabilities [2]. These risks may begin before placement and can continue throughout the period in care and beyond [2].

When compared to other children, youth in foster care consistently exhibit greater rates of depressive disorders, with a range of possible causes, including genetic, environmental, and systemic factors [2]. For example, they tend to have higher rates of child abuse and neglect [4]. A history of adverse childhood experiences (ACE) can increase vulnerability to developing depressive disorders [5]. Additionally, they may also have fewer resources to access mental health services [2].

Unfortunately, there is limited information on the prevalence of mental health disorders among youth in foster care [4]. This population is often underrepresented in epidemiological studies because frequent placement changes and challenges related to consent make research participation difficult [4].

This narrative review will synthesize the current understanding of depressive disorders in foster care, including demographic factors, protective factors, impact on education, and screening challenges in foster youth. We hope to help increase awareness and the ability of care providers to detect, prevent, and manage depressive disorders in foster care.

## Review

Methods

A literature search was conducted using online databases, including PubMed and Google Scholar. The exclusion criteria included studies published before 2015. Keywords used in search engines included “depression,” “major depressive disorder,” “foster children,” “foster care,” and “mental health.” All articles published in English from 2015 to 2020 were reviewed, with priority given to meta-analyses, systematic reviews, cohort studies, and high-impact primary research. All articles were required to address epidemiology, risk factors, mechanisms, treatments, and/or outcomes of depression in children in foster care.

Epidemiology of depression in foster care

Multiple studies demonstrate significantly increased prevalence rates of depressive disorders in children and adolescents within foster care compared to the general pediatric population. In addition, studies conducted in several countries have shown an increased risk of mental illness and suicide among children in foster care [2]. Furthermore, when examining children in foster care, the most common types of mental illnesses found in this population are oppositional defiant disorder/conduct disorder, major depressive disorder, post-traumatic stress disorder (PTSD), and reactive attachment disorder [1]. Although this study focuses on depressive disorders, many other mental illnesses exist in this population of children. In fact, of the 3,104 children in foster care, 49% had a mental health disorder, and 11% had a depressive disorder [4]. Unfortunately, these concerns have been found to continue into adulthood. One study examined over 600,000 adult patients with mental disorders and found that 30% had a prior history of living in foster care [3].

Additionally, further research shows that gender and age are further divided within the population. Girls in the foster care system tend to experience increased rates of depression compared to their male counterparts [2]. For example, a study using the Stockholm School Survey, a survey that monitors living conditions, found that girls in foster care reported higher rates of depression and had reported “rarely enjoying life” more often [2]. Many of the foster children reported that depression symptoms interfered with their quality of sleep [2]. Additionally, rates of depression are increased across all age groups, but peak during adolescence [2].

Numerous studies have attempted to identify the causes of these vast disparities between genders and ages; however, multiple factors continue to influence the overall cause of these disparities. Prior to the time that children are placed into foster homes, nearly all children have been victims of some form of maltreatment, whether physical or emotional abuse, psychogenic issues, and/or social or economic issues [2-4]. For these reasons and more, depression in foster children continues to be a major problem that needs to be addressed.

Early adverse experiences

It is no surprise that maltreatment and psychosocial concerns can play a role in the development of depression. The ACE framework provides a significant explanation of how cumulative adversity affects the development of the brain, increasing the risk of developing depression. For example, a study analyzed the effects of parental ACEs on adolescent depression [5]. It was found, through an analysis of the Longitudinal Study of Australian Children, that the presence of two or more ACEs, which included some aspect of deprivation, significantly increased the risk of depression across all age groups [5]. Although foster children are among those who generally have a higher incidence of ACE history, the results of this study support the premise that children with any history of ACEs have the potential to have a higher risk of developing depression [3,5]. Early maltreatment and neglect, as well as exposure to parental psychiatric illness, were among the major precursors for later childhood depressive symptoms. Another study confirmed that all types of maltreatment strongly correlate with depression [3,5]. The results of this study also indicate that the combination of multiple types of maltreatment presents a substantially increased risk for developing depressive conditions [5].

Demographic and genetic moderators

Teenage foster youth, girls, and children with a family history of psychiatric illness and heritable substance dependency are at especially elevated risk [6]. One study found that White adolescents were more likely to be diagnosed with a mental health condition, such as major depressive disorder, before entering foster care, while minority groups were more likely to be diagnosed with a mental health condition during foster care [6]. Further research is needed to explore how systemic factors, including racial bias and access to mental health services, influence disparities in mental health identification and treatment among foster children across different demographic groups.

Protective and buffering factors

Fostering an environment with stable and supportive foster care placements, connecting to a therapist through a strong therapeutic alliance, and using a trauma-informed care model can greatly mitigate the risk of depression for youth in the foster care system [3,7]. It is also critically important to provide social support and to intervene early in the youth's mental health struggles. Studies have shown that aging out of foster care, with little to no social support during the transition, tremendously impacts mental health outcomes [7].

Truthfully, the prevalence of depression in foster children far exceeds that of non-fostered peers. It is often comorbid with anxiety, PTSD, behavioral difficulties, and attachment disorders [1]. When youth experience chronic depression, academic performance declines, suicidal ideation increases, placement breakdown occurs, and the youth is at risk of experiencing poor transition-into-adulthood outcomes [1,2]. Depression has been linked to academic underperformance, unemployment, substance use, teenage pregnancy, and more [7-9].

Family and placement characteristics

Apart from early adversity, another important factor regarding depression outcomes in foster children lies in the quality of their foster home environment. Studies focusing on various aspects of foster families have indicated that, overall, more positive features of care are associated with lower levels of internalizing symptoms, such as depression [10]. Positive features of care may include consistency and emotional support. Family environments that are more conflict-ridden, inconsistent, and emotionally detached are associated with more depressive symptoms in children [10]. Even postplacement relationships have the potential to alter mental health in these young individuals. This further supports the call for family-based interventions that educate caregivers to effectively help facilitate emotional recovery in foster children.

Placement instability and emotional health

Placement instability reflects a foster care-specific stressor with significant emotional consequences. Youth experiencing repeated placement disruptions exhibit significantly elevated rates of depressive symptoms. Studies show high rates of symptoms, such as emotional withdrawal, hopelessness, and anhedonia [11]. Recent longitudinal studies confirm that any additional placement change exacerbates emotional distress and interferes with attachment formation, a crucial part of child development [11]. Instability itself has been found to be an independent predictor of depression. These findings suggest that social welfare is a crucial part of mental health intervention.

Educational disruption and impact on depression

Educational instability is another overlooked contributor to depression unique to foster children. Many foster children face multiple educational disruptions, including school changes, academic delays, and unmet special education needs. Additionally, distractions at home make focusing on school especially difficult. Foster children often experience a more difficult time forming relationships with their peers and engaging in support systems offered at school. Studies show that academic disengagement exacerbates underlying depression [12]. One point of intervention is academic support programs that can help address depression in this population.

Caregiver mental health and relational context

Another important influence to consider in the emotional outcome of foster children is the mental health of their caregivers. Current research supports an association between mental health in foster children and their caregivers [13]. In fact, caregiver stress, burnout, and depression can impact the emotional state of their foster children, as evidenced by higher levels of internalized symptoms among the children [13]. Interestingly, mental health support for the caregivers is linked to improved emotional regulation in the children they care for [13]. This supports the relational nature of depression in the foster child.

Psychosocial interventions

Trauma-focused cognitive behavioral therapy (TF-CBT) and interpersonal therapy have demonstrated efficacy in alleviating depressive symptoms among foster youth [3]. Attachment-focused and family-based interventions, though under-researched, are well supported in the clinical environment. By educating about depression, especially during the transition year of aging out of foster care, we can address prevention and management [7,13,14]. Collaborative care, mental health integration, and trauma-informed policy reforms demonstrate potential for improving detection and outcomes [3,8,15,16].

Screening and detection challenges

Despite there being a clear concern of depression in foster children, routine screening for such has had its challenges. Multiple reviews on depression in primary healthcare suggest that depression is not as widely screened for in the child welfare system. Some of the obstacles to depression detection in foster children include a lack of placement stability, a lack of integration between child welfare and healthcare services, and a lack of training for healthcare workers in trauma-focused care [17]. In addition, depressive symptoms are frequently masked by externalizing behaviors, trauma-related responses, or adjustment issues [18-20]. This makes it much easier for depression to be mistakenly misattributed to something else. Lastly, foster youth may underreport depressive symptoms in a depression screening, such as a PHQ-9, due to fear of stigma, trust issues, or beliefs that it can negatively impact their placement in foster care. One way to reduce disparities in detecting depression in this population is the use of standardized screening measures at multiple time points throughout their foster care placement.

## Conclusions

Children in foster care are at disproportionate risk of depression due to layers of adversity and system challenges. While recent research clarifies prevalence and pathogenesis, considerable barriers remain regarding detection, timely intervention, and prevention. Routine screening, trauma-informed interventions, and policy reform are essential in addressing psychiatric disparities in this population. There are still limited large-scale trials on preventative interventions, a lack of standardized protocols for depression screening across jurisdictions, and insufficient long-term outcome data.

Further research is necessary to clarify best practices and develop child-centered care models. Our recommendations are to educate foster youth, especially those transitioning out of foster care, about depression in all forms. We also note the need for further research on racial and ethnic disparities when obtaining a diagnosis of major depressive disorder as a foster child.
